# Bacterial Diversity at Himalayan Pink Salt Extraction Site

**DOI:** 10.3390/biology14030316

**Published:** 2025-03-20

**Authors:** Yasmeen Malik, Imran Ali, Ashif Sajjad, Luhuai Jing, Irfana Iqbal, Atiq ur Rehman, Toquier Azam, Xiaoming Chen

**Affiliations:** 1 School of Life Science and Engineering, Southwest University of Science and Technology, Mianyang 621010, China; yasmeenmalik2012adc@gmail.com (Y.M.); irfana.ali@outlook.com (I.I.); toquierazam@gmail.com (T.A.); 2 Institute of Biochemistry, University of Balochistan, Quetta 87300, Pakistan; ashifsajjad@hotmail.com; 3 Mines and Minerals Development Department, Government of Balochistan, Quetta 87300, Pakistan; atiqshahwani@gmail.com; 4 Institute of Molecular Biology and Biotechnology, University of Lahore, Lahore 54600, Pakistan; 5 College of Life Sciences, Sichuan University, Chengdu 610064, China; jingluhuai@163.com

**Keywords:** metagenomics, extremophiles, halophilic, pink salt, hypersaline environments

## Abstract

Khewra Salt Mine, the second-largest salt deposit in the world, is located in the Kohistan Salt Range, Pakistan. Parameters such as pH, salinity, and trace minerals were assessed, offering concise insights into the Khewra Salt Mine ecosystem. This study discloses soil moisture content in Khewra ranging from 8.27% to 26.59%, with soil pH levels between 7.9 and 8.65, while brine maintains a close-to-neutral pH of 6.8–7.5. Soil samples exhibit higher salinity (30–36%) than brine (29–33%), with soil containing 29.8 wt% Na and 45.3 wt% Cl, while K, Fe, Mg, and SO_4_ ions were found comparatively higher in soil. Conversely, Ca ion content was higher in brine. Organic matter, total organic carbon, and nitrogen content were lower in brine than in soil. Metagenomics techniques, including 16S rRNA amplicon sequencing, uncovered the microbial diversity of the Khewra Salt Mine. Biodiversity analysis revealed 17 phyla, with Firmicutes (35.5%) and Bacteroidetes (26.5%) dominating soil. Proteobacteria (23.3%) and Euryarchaeota (10.6%) were more abundant in brine. The top ten genera contributed up to 75% of total diversity, with Lactobacillus (33.7%) and Flexispira (9.1%) prominent in soil. Halobacteria members Halorhabdus (5.0%) and Haloarcula (2.2%) were exclusive to saltwater. Alpha diversity was higher in salt, indicating distinct microbial communities between soil and water. Fascinating interconnections between physical factors and microbial diversity in the Khewra Salt Mine were noted. The microbial diversity in the Khewra Salt Mine was strongly influenced by physical factors, particularly pH (6.8–8.65) and salinity (29.9–36%). Higher salinity and alkaline pH in soil were associated with dominant populations of Firmicutes and Bacteroidetes, while brine samples with near-neutral pH supported greater abundances of Proteobacteria and Euryarchaeota, highlighting distinct microbial community compositions between the two environments. Salinity (29.90 to 36.0%) and pH (6.8 to 8.65) varied; however, microbial abundance was positively connected with all other physical parameters. Microbial abundance was found to increase as key physical conditions, such as salinity (29.90 to 36.0%) and pH (6.8 to 8.65), improved, indicating a positive correlation between microbial growth and these environmental factors. The findings suggest that the Khewra Salt Mine hold promise for future research uncovering microorganisms with significant food, pharmaceutical, and biotechnological potential.

## Simple Summary

This study focuses on the diverse microbial life in the Khewra Salt Mine, one of the largest salt mines in the world, located in Pakistan. This research aims to explore the types of bacteria and other microorganisms living in the mine’s soil and brine, which are exposed to extreme conditions such as high salinity and alkaline pH. Using advanced genetic techniques, we discovered a wide variety of microbes, with two main groups of bacteria thriving in different environments, one in the soil and the other in the salty brine. The soil had more diverse bacteria like *Lactobacillus*, while the brine supported different groups, including halophiles that thrive in extreme salt conditions. This study shows that these microbes are well-adapted to their harsh surroundings and could have important applications in food preservation, biotechnology, and medicine. By understanding how these organisms survive in such conditions, researchers can learn more about the potential for similar life forms on other planets, as well as how to use these microbes in industrial applications.

## 1. Introduction

Table salt, or sodium chloride, is extensively utilized in the culinary business as a flavoring agent, texture garnishing [1], preservative, and food protective [2]. Saline environments such as sun ponds, saline lakes, brine springs, rock salts, and saltwater are the source of commercial table salt [3]. Before being distributed, most marketable salts are purified and finely milled [4]. Additionally, saline settings can give rise to halophiles, bacteria that love salt [5]. Halophiles are recognized to be a unique class of extremophiles with extreme salinity requisite, including entities of the “Three Domains Classification” [6]. Furthermore, these microbes are crucial for usage in the biomedical industry because of their distinct metabolism, minimal food requirements, and capacity to adapt to hostile environments.

Salt mines are the primary source of rock salt worldwide, including the Khewra Salt Mine in Punjab, Pakistan, which is the world’s second largest salt mine, producing the finest pink salt [7]. The presence of viable microbes within fluid inclusions in halite crystals has been substantiated by several studies. During the crystallization process, these bacteria may become trapped in the fluid inclusions and endure till the process of extraction and packaging. The proteome of *Halobacterium salinarum* cells was examined after two months being entrapped in halite brine inclusions. The results showed a high degree of similarity to stationary phase liquid cultures, with specific down-regulation of ribosomal proteins, indicating acclimation to the halite environment [8]. Similarly, research highlighted the resilience of extremely halophilic archaeal communities in crystallizer ponds and halite crystals. The carotenoid-rich microbial community positively influences salt production and yield by enhancing light absorption and increasing local temperature [9].

According to a recent study [10], microbes typically inhabit evaporative formations. Microbes in halite crystals proved to be the best models for studying the patterns of evolution in ancient microorganisms because the experiments serve to authenticate results such as surface sterilization, first crystallization, in situ hyalinization observations, and adequate repeatability due to their unique biodiversity [11]. Halophiles have established diverse modifications to manage within the saline atmosphere [12]. Using the “salt in” approach [13], the existence of K^+^ and, comparatively less regularly, Na^+^ is central in balancing the intracellular osmotic pressure of the microbe with that of the atmosphere [14]. Furthermore, some halophiles gather suitable organic solutes in the cytoplasm to control the intracellular osmotic pressure [12]. Aside from that, the most extreme halophiles are Archaea, which frequently have optimal salt needs of more than 25% *w*/*v* dissolved salts [15]. In this situation, this study was designed to study the hypersaline ecosystems and their habitats, specifically to understand the available physical factors, raw quality of pink salt, and 16S RNA diversity of the microbes, specifically halophilic archaea and bacteria from Pakistan’s Khewra Salt Mine. The current study focused on microbial diversity in soil and brine samples from the Khewra Salt Mine and their environmental relevance to extremophilic life forms [16].

Halophiles, especially halophilic bacteria, can be found in table salt and excessively salted meals. Some of these bacteria may have started in the original salt-harvesting region. Therefore, identifying the species colonizing salt crystals contributes to our knowledge of the mechanisms governing microbial survival in harsh environments as well as the processes of evolution [17]. In light of this, as long as the salt content is high, manufactured food can be considered a haven for halophiles [18]. Certainly, halophiles are also present in fermented seafood [19], cheeses [20], sauces [21], pickles [22], and green table olives where table salt and spices are used as preservatives [23].

All three types of salt (lake salt, sea salt, and rock salt) found in lakes, seas, and rocks are abundant in Pakistan. Soda lakes, salt lakes, and rock salt are examples of the hypersaline settings that can be found nationwide. The earliest salt deposits in the Asian subcontinent are found in this salt range, including rock formations from the pre-Cambrian to the most recent geological periods [24]. According to research [25], the bacteria contained ancient DNA sequences that might have been retained in their salt crystals that would eventually reflect Eocene life in light of the claim that halophiles are the initiators of the terrestrial ecosystems [26]. The nature of these organisms and their identities are still unknown. Numerous investigations are being conducted on halotolerant enzymes produced by bacteria and culture-dependent bacterial isolations related to salt mines [27]. This study investigates enzymes from salt marsh ecosystems, highlighting their biotechnological potential in ionic environments [28]. Similarly, research focuses on the diversity of halotolerant and halophilic bacteria in the Karak Salt Mine and the Khewra Salt Mine, emphasizing their potential for enzyme production [29,30]. With the availability of next-generation sequencing, metagenomics investigations are being efficiently employed to investigate the variety of microbes in harsh environments [31]. According to a recent study, it is proving to be highly beneficial in reconstructing the draught and the sequences of the closed genome, uncultivated bacteria, which are utilized to elaborate the microbial makeup and population in different hypersaline environments [32].

The metagenomics analysis makes it evident how microbial communities interact with their surroundings and demonstrate the diversity of their ecology. There are minimal reports on the microbiota of Himalayan pink salt, and those that do exist typically concentrate on the archaeal and bacterial taxa. Nevertheless, metagenomic studies represent the processes of communication among environment and evolution and the importance of microbes in nutrient cycling [33]. The current study uses Illumina 16S rRNA amplicon sequencing and metagenomic analysis to fully characterize the bacterial diversity of the two separate groups of the described location (soil and brine) and all abiotic conditions. To better understand the distribution of both archaeal and bacterial isolates, microbes were isolated from soil and brine samples using standard culturing techniques. The results revealed distinct microbial populations in each sample type, with certain archaeal species predominantly found in the brine, while bacterial isolates, including *Lactobacillus* and *Flexispira*, were more abundant in the soil samples, their relationships with physical factors, and the presence of pathogenic and non-pathogenic species in raw salt halite concerning human health were determined. We have examined the microbial contents of the two distinct groups, three soil samples, and three brine samples from six different locations of the Khewra Salt Mine, the source of Himalayan pink salt.

## 2. Materials and Methods

### 2.1. Khewra Salt Mine

**Figure 1:**
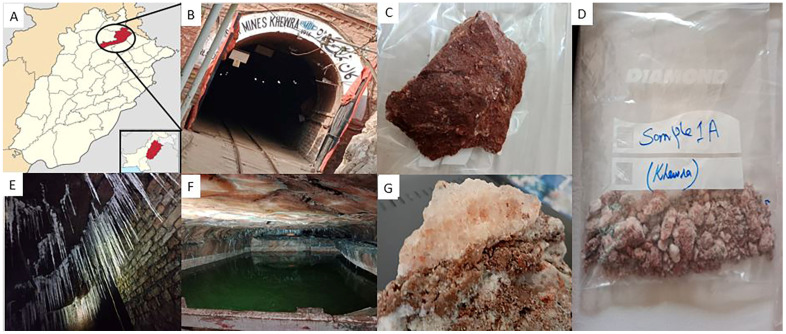
(**A**) Map displaying the location of Khewra Salt Mine in district Jhelum, Punjab, Pakistan. (**B**) Main entrance of Khewra Salt Mine. (**C**,**D**) Samples taken from the mine walls that were made of brownish saline soil and pink-colored halite. (**E**) Needle-like salt crystals due to brine dripping from the roof of Khewra Salt Mine. (**F**) Saturated brine pool flowing inside the salt mine. (**G**) Pinkish-white salt along brownish soil sample from the Khewra Salt Mine.

**Figure 2:**
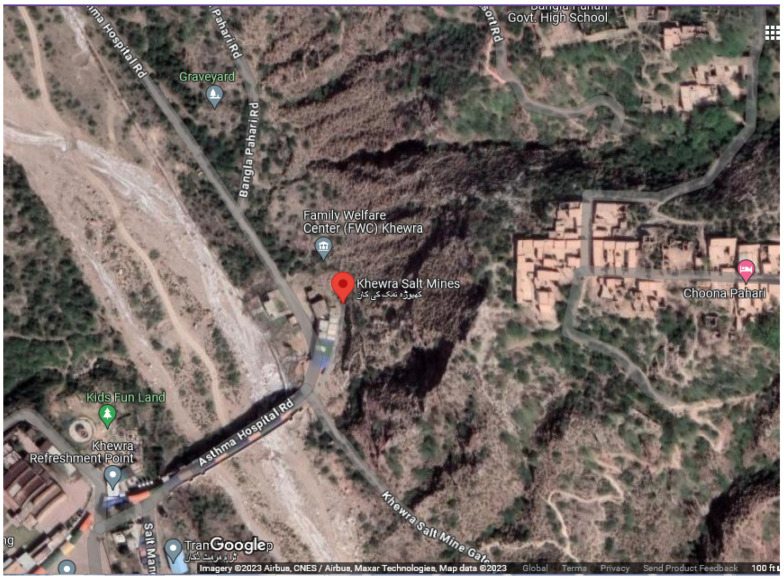
Google Earth Map showing the sampling site and neighboring location at Jhelum district, Punjab province, Pakistan (Imagery© 2023 Airbus, CNES/Airbus, Maxar Technologies, Map data © 2023 google).

Salt was discovered at Khewra during Alexander’s visit to South Asia [34]. The Khewra Salt Mine is located in the Jhelum District, 160 km south of Islamabad, the capital of Pakistan. This mine is located at the foot of the Salt Mountains and is one of the oldest in the world. The latitude and longitude of the study area are 32°55′ N and 73°14′ E, respectively, and the geographical location of the Khewra Salt Mine is depicted in Figure 1A, while sampling sites and surrounding areas are shown on the map in Figure 2. The Khewra Salt Mine has a total salt deposit of 220 million tons, making it the second-largest salt reserve in the world. The mine’s current annual production is 465,000 tons. Khewra mine salt is transparent, white, pink, and reddish to beef-red. The diverse colors of Khewra Salt Mine’s salt are a result of the interplay between mineral impurities, particularly iron oxides, and the geological and environmental conditions during the salt’s formation. These factors collectively contribute to the unique and varied hues observed in the mined salt. The reddish and beef-red hues in the salt are largely attributed to the presence of iron oxides. These compounds impart a red to reddish-brown color to the salt crystals. The concentration of iron impurities varies within different layers of the mine, leading to the observed color differences [35]. Beyond iron, other trace minerals such as calcium, magnesium, potassium, copper, and zinc are present in the salt deposits. These minerals contribute to the pink and reddish tones of the salt. The specific mineral composition of each salt layer influences its exact coloration. At these points, rock salt consists of 98% pure NaCl [35].

### 2.2. Sample Collection

Samples of brine and salt were gathered from two distinguished selected sites in the Khewra Salt Mine under favorable conditions that prevent the introduction of exogenous microbial contamination. The in situ temperature inside the Khewra Salt Mine remained consistently between 18 and 20 °C throughout the sampling. Salt crystals were removed from the mine walls using a sterile spatula and placed in sterile, sealed polyethylene bags for storage. Meanwhile, brine dripping from mine’s wall efflorescence and saturated brine water samples from pools were collected in sterile sampling bottles and preserved at −4 °C before further examination. Each sample was sealed and moved to the laboratory (University of Balochistan, Quetta) within hours to −4 °C. Coordinates for each sampling location on GPS were noted. The temperature and pH value of the brine were measured using pH paper at sampling points and then using a pH meter under laboratory conditions (Sartorius professional meter PP15, Sartorius, Göttingen, Germany).

Salinity testing was performed and calculated with a handheld refractometer, and the total amount of dissolved solids was also noted. Chemical analysis of major ions (sulfate, chloride, phosphate, bicarbonate) in brine was done by using Spectroquant (Pharo 100 SpectroquantR Merck, Darmstadt, Germany) under the direction of the American Public Health Association’s (APHA) established methods for the analysis of water and wastewater.

### 2.3. Chemicals and Reagents

The analytical-grade reagents and chemicals utilized in this investigation were procured from AVACADO Research Chemicals Ltd., Sigma Aldrich (St. Louis, MA, USA), E Merck (Darmstadt, Germany), Difco Laboratories (Detroit, Frankin Lakes, NJ, USA), among others (London, UK).

### 2.4. Physicochemical Examination of Soil and Brine Samples

To prepare soil suspensions for physicochemical analysis, 10 g of soil sample was mixed with 50 mL of distilled water. The salinity and pH of soil suspensions were determined using a calibrated salinity refractometer and pH meter, respectively. Moisture content was calculated by comparing the weights of fresh and dried soil samples. Organic matter and total organic carbon (TOC) were measured using the Walkley–Black wet combustion method and the Teckmar-Dohrmann Phoenix 8000 analyzer (Teledyne Tekmer, Mason, OH, USA), respectively [36]. The total nitrogen was measured using the Kjeldahl digestion method with a Tecator DS-6 Digester (TN) (Foss, Stockholm, Sweden) [37].

### 2.5. Isolation of Halophilic Microorganisms

Standard isolation techniques mentioned in our previous publications have been implemented with few required modifications [38]. Genomic DNA was extracted using the NucleoSpin Viral DNA Extraction Kit (Macherey-Nagel, Duren, Germany) following the manufacturer’s protocol [39]. DNA quality was confirmed by 1% agarose gel electrophoresis in 0.5× TBE buffer (pH 7.4) and visualized using a Bio-Rad Gel Documentation System (Bio-Rad, Hercules, CA, USA). The purified DNA was maintained at −20 °C until it was required for further processing.

### 2.6. 16S rRNA Amplicon Sequencing

Isolated DNA was transported to Macrogen (Seoul, Republic of Korea) for 16S rRNA metagenomic sequencing. The polymerase chain reaction was used to amplify the V3–V4 hypervariable region of the 16S rRNA gene using primers 341F (5′-CCTAYGGGRBGCASCAG-3′) and 806R (5′-GGACTACNNGGGTATCTAAT-3′) with barcodes.

PCR reactions contained 25 µL Phusion^®^ High-Fidelity PCR Master Mix (New England Biolabs, Ipswich, MA, USA), 25 pmol of each primer, 1 µL DMSO (100%), 0.25 µL dNTPs (10 mM), 0.25 µL Taq polymerase, 2.5 µL 10× buffer, and 2 µL DNA template. The denaturation stage of the PCR protocol is crucial and lasts for five minutes at 94 °C. Subsequently, thirty-four cycles of primer annealing at 55 °C for forty-five seconds, denaturation at 94 °C for thirty seconds, extension at 72 °C for two minutes, and a final extension phase at 72 °C for five minutes were performed. The PCR products were mixed with an equivalent volume of 1x loading buffer which contained SYBR green, and subsequently electrophoresed on a 2% agarose gel to determine its identity. Samples with a prominent main band between 400 and 470 bp were selected for further testing. Macrogene’s IonS5TMXL platform (Thermofisher, Waltham, MA, USA) was employed to sequence the libraries generated by the Ion Plus Fragment Library Kit (Macrogen, Seoul, Republic of Korea).

### 2.7. Sequence Analysis

Raw 16S rRNA paired-end reads were demultiplexed and preprocessed by Macrogen (Republic of Korea). Quantitative Insights into Microbial Ecology (QIIME ver. 2.2020.6) was employed to identify these leads [40]. The q2 manifest file import technique was employed to import paired-ended FASTQ reads into QIIME, and the q2 Dada2 denoising method was employed to filter the quality [41]. Frequencies of amplicon sequence variants (ASVs) and their corresponding classical sequence lists were produced using the QIIME program. To assign a classifier to a typical ASV sequence, q2-feature-classifier was used [42]. A machine learning technique ‘classify-sklearn’ using a basic Bayesian classifier was used on the Silva 138_release 99% OTU reference sequence [43].

### 2.8. Statistical Analysis

We used R (www.r-project.org, accessed on 24 November 2024) software for downstream analysis and graphics drawing. Four metrics were calculated to estimate the alpha diversity (within the sample). Shannon (a quantitative part of the number of OTUs present in a given sample and their relative abundance or community richness), noticed OTUs (the number of OTUs present in a given sample), Chao1 (estimated OTU wealth identified), and Simpson’s metric (dominance, measure of diversity) were used. Alpha diversity indices were correlated using the nonparametric Kruskal–Wallis test. Distance metrics were calculated for beta (between-sample) diversity, as well as unweighted values [44,45] and weighted UniFrac (a qualitative measure of community dissimilarity that incorporates phylogenetic relationships between the features) [46]. We visualized beta diversity relationships using principal component analysis (PCA). Beta diversity metrics were analyzed using a permutation ANOVA test (PERMANOVA) [47].

## 3. Results

### 3.1. Physiochemical Analysis

The results of the physiochemical analysis of soil samples are summarized in Table 1, and brine samples in Table 2. The color of the soil ranged from pink to brown. A temperature of 29.9–33 °C was observed. The pH of the brine was close to neutral, between 6.8 and 7.5, whereas the soil samples were alkaline or slightly alkaline, having a pH of 7.9–8.65. Salinity was high in soil samples, ranging from 30 to 36%, compared to brine, which runs from 29 to 33%. The pH values observed in the Khewra Salt Mine ranged from 6.8 to 8.65, with brine samples maintaining a near-neutral pH (6.8–7.5) and soil samples exhibiting slightly alkaline conditions (7.9–8.65). These pH ranges are consistent with other hypersaline environments such as the Dead Sea (pH 6–8) and the Great Salt Plains (pH 7–9). However, the stable near-neutral pH of brine in Khewra is notable, as many hypersaline lakes and ponds exhibit greater variability, potentially influencing microbial diversity and resilience. This highlights Khewra’s suitability for supporting both halophilic and halotolerant microorganisms with distinct ecological adaptations. Soil samples enclose about 29.8 wt% Na and 45.3 wt% Cl with a slight difference in brine. Comparatively, the wt% of K, Fe, Mg, and SO_4_ ions were higher in soil than in brine. In contrast, the wt% of Ca ion was higher in brine than in the soil samples. Nitrogen content, total organic carbon, and organic matter were lower in brine than in soil.

### 3.2. Taxonomic Analysis of Bacterial Diversity

**Figure 3:**
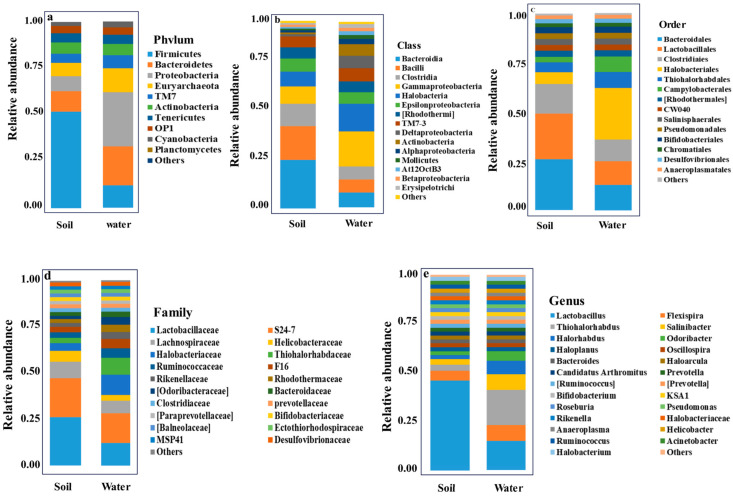
(**a**) Taxa bar plots of bacterial diversity at phylum level in two different groups. (**b**) Taxa bar plots of bacterial diversity at class level in two different groups. (**c**) Taxa bar plots of bacterial diversity at order level in two different groups. (**d**) Taxa bar plots of bacterial diversity at family level in two different groups. (**e**) Taxa bar plots of bacterial diversity at genus level in two different groups.

Figure 3 shows the bacterial analysis of the soil and brine water samples from six different spots of Khewra Salt Mine. The data showed a diverse microbiome profile in the soil and brine samples of the study subjects. The soil and brine microbiome were assessed at phylum, class, order, family, and genus levels.

According to the taxonomy assignment at the phylum level, 17 distinct phyla with varying relative abundance were seen in the soil and water groups (Figure 3). The top five abundant phyla provided up to 98% of the overall diversity. Interestingly, Firmicutes (35.5%) continued to be the most common group, with Bacteroidetes (26.5%), Proteobacteria (23.3%), and Euryarchaeota (10.6%) following closely behind. Candidate division TM7, Actinobacteria, Tenericutes, OP1, Cyanobacteria, and Planctomycetes accounted for a relatively small portion of the total diversity. Bacteroidetes and the phylum Firmicutes were present in both samples. Additionally, we discovered that the soil had comparatively more Firmicutes and Bacteroidetes. Proteobacteria are more prevalent in salt water, and Euryarchaeota can only be found there. Up to 75% of the total diversity was supplied by the 10 most abundant genera at the genus level. They were notably having Salinibacter (5.6%), Odoribacter (3.9%), Oscillospira (2.7%), Thiohalorhabdus (8.5%), Lactobacillus (33.7%), and Bacteroides (2.6%). The two species of Halobacteria with the highest abundance in our sample were Haloarcula (5.0%) and Halorhabdus (2.2%) and were limited to samples of salt water.

### 3.3. Alpha and Beta Diversity Analysis

**Figure 4:**
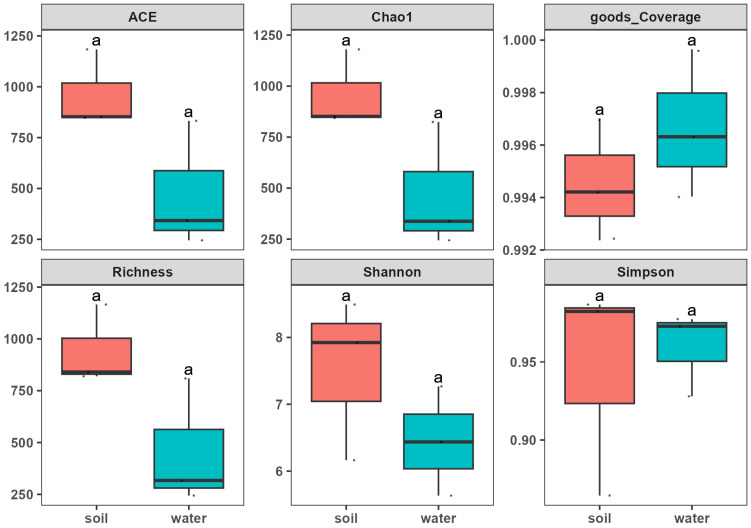
Alpha diversity indices in soil (red) and brine water (blue) microbial diversity. ACE, Chao1, Richness, and Shannon are higher in soil than salt water. The ‘a’ represents statistical significance, while ‘.’ represents outliers.

Microbial communities in soil may have higher alpha diversity. Our results showed that soil microbial community alpha index, such as ACE, Chao1, Richness, and Shannon, is higher than in salt water (Figure 4). However, the results were not significant since we only had three replications.

**Figure 5:**
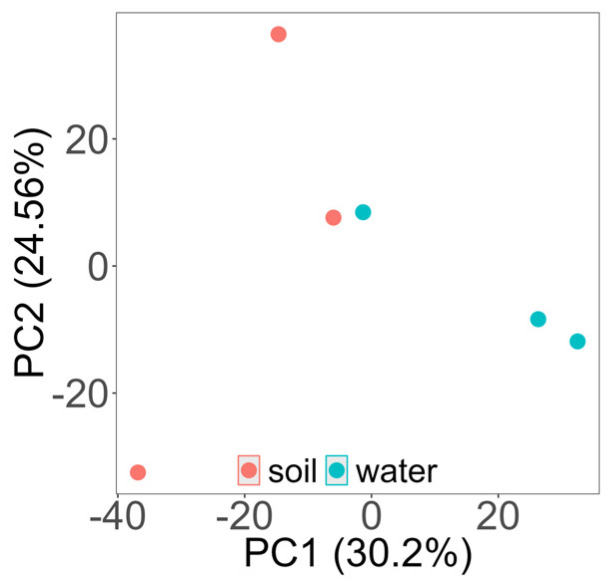
Beta diversity indices showing the distribution of samples from two different groups.

Based on PCA analysis of microbial beta diversity, we found differences in microbial community structures in soil and saline water (Figure 5). Sample points representing water microbial communities clustered better, indicating that the differences in microbial communities in water were minor compared to soil ones. We can see that the microbial community in salt water differs from that in soil.

The higher alpha diversity indices (Shannon and Chao1) in soil suggest that alkaline and highly saline conditions create niches for a broader range of halotolerant species compared to brine, where conditions are more stable and selective. The beta diversity analysis highlights distinct clustering between soil and brine samples, reinforcing the notion that pH and salinity gradients shape unique microbial communities. These environmental differences likely act as ecological filters, allowing certain taxa to dominate in specific conditions. The clustering patterns in beta diversity directly reflect how microbial compositions are structured along the salinity and pH gradients, with organisms requiring specific adaptations to thrive under varying levels of ionic stress and hydrogen ion concentration.

## 4. Discussion

This study marks a pioneering investigation into the microbial diversity of the Khewra Salt Mine, utilizing metagenomics and physicochemical analyses to reveal the intricate relationship between halophilic microbial communities and their extreme environment. Unlike previous studies, which primarily focused on isolated aspects of salt mine ecosystems, our research provides a comprehensive profile of microbial diversity and its direct correlation with environmental factors such as salinity, pH, and ion composition. By uncovering unidentified microbial genera and demonstrating the resilience of halophiles in fluid inclusions, this work not only enhances our understanding of extremophiles, but also contributes to practical applications in salt production, preservation, and biotechnological development. The findings highlight the potential role of microbial communities in influencing salt quality, production efficiency, and preservation mechanisms, offering insights into evolutionary processes and microbial survival strategies that can be leveraged in industrial and food technology contexts.

Most processed foods contain sodium chloride as a preservative, significantly impacting osmotic pressure [48]. Sodium and chloride can interact with water molecules during the salting process, reducing the water activity of the food product [49]. Consequently, salinity can stop microbial spoilage [50]. Salt limits aerobic growth by lowering oxygen solubility. The enzymatic activity of certain microbes can also be inhibited by high salt and chloride concentrations [51]. In the manifestation of salt, microbial cells expend more energy by pumping sodium out of their cells to deal with the severe influence of osmotic shock [52]. These halophiles are also highly desirable candidates for their utilization in biotechnology since they are employed to produce active biomolecules and medicinal applications [31]. For this reason, the extreme environments (the Pre-Rif region in Morocco and the Dead Sea in Jordan) were explored [53,54] for the isolation of bacteria of biotechnological potential and [55] were the first to identify the antimicrobial agents of Actinobacteria found in the Ahouli-Mibladen-Zaida mining sites. Likewise, we explored *Actinobacteria* in a minor percentage from Khewra Salt Mine. The potential for Actinobacteria to produce antibiotics was considered as a future research direction, as Actinobacteria are known for their biotechnological significance. The existence of this phylum, which is highly beneficial for the synthesis of antibiotics [56], is demonstrated by our findings. Certainly, research to generate novel treatments is urgently needed in an era of multidrug-resistant microbes, rising cancer rates, and extreme environmental degradation [57]. Numerous valuable substances, such as ectoines, biosurfactants, and antibacterial medicines like streptomycin, are isolated from halophiles [58] and have been used in biotechnology, cosmetics, and medicine [59]. Studying the organisms confined in the enclosed space of salt mines is crucial. Microbes found in salt mines may impact the preservation of life in the fluid trapped in the salt crystals at hypersalinity [60,61]. Numerous salt environments, including plains, rock salts, food-grade salts, and salt mines, have been shown to contain active and ancient microorganisms [62,63,64,65].

In this study, we identified a diverse microbial community in the soil and brine samples from the Khewra Salt Mine. These microbial communities are strongly influenced by the extreme environmental conditions present in these habitats, such as high salinity and alkaline pH. The results shed light on the resilience of halophiles and other extremophiles in these environments, furthering our understanding of microbial life in hypersaline ecosystems.

In addition to the microbial diversity observed, the soil and brine samples from the Khewra Salt Mine contain salts with significant mineral content, including sodium, chloride, calcium, and magnesium. Although these samples are not marketed directly for commercial use, the salt present in these environments shares similarities with the Himalayan pink salt produced by the mine. This salt, known for its trace minerals, plays a crucial role in the nutritional profile of Khewra Salt, commonly used in culinary applications and health supplements. The microbial communities identified in these environments may contribute to understanding the microbial ecosystems in salt production and their potential impact on the quality and nutritional aspects of the commercial salt

Our investigation of the microbiota of the Khewra Salt Mine is especially significant because of the site’s historical and natural value and the significance of Himalayan pink salt. The Khewra Salt Mine is a prime example of a hypersaline site, and its analysis may help us better understand the ecology and genomics of halophiles. Using metagenomics, our results offer a fresh perspective on the relative abundance of the microbiota of the Khewra Salt Mine. This perspective sheds light on the microbiome’s diversity, distribution, and functionality and its relationship to physicochemical variables. Therefore, this study is the first to use metagenomics to investigate the halophiles’ earlier unreported metagenomic community profiles and correlate them with their physicochemical parameters and the native microbiota of the Khewra Salt Mine [7].

The fundamental physicochemical characteristics of the brine and soil under study were most likely only marginally correlated with the observed variations in the makeup of the microbial communities. This is because there is very little difference in the samples between the two groups, and the soil and brine in the Khewra Salt Mine have nearly identical salinity, pH, and primary ion levels. The soil environment, characterized by higher salinity (30–36%) and alkaline pH (7.9–8.65), supports a microbial community dominated by halotolerant and alkaline-adapted phyla such as Firmicutes (35.5%) and Bacteroidetes (26.5%). These organisms are well-suited to high-salinity conditions because they employ osmoregulatory mechanisms, such as accumulating compatible solutes like potassium and synthesizing salt-stable enzymes [66]. Conversely, the brine samples, with their slightly lower salinity (29.9–33%) and near-neutral pH (6.8–7.5), favor taxa such as Proteobacteria (23.3%) and Euryarchaeota (10.6%). These organisms thrive in less alkaline conditions and exhibit a higher tolerance for ionic exchange [67]. Additionally, the article “Evaluation of Microbial Assemblages in Various Saline-Alkaline Soils Driven by Soluble Salt Ion Components” (2022) explores how microbial communities in saline-alkaline soils adapt to high salinity and alkalinity by regulating ion homeostasis and synthesizing compatible solutes [68]. By Correlating these differences, the alignment of physiological adaptations was observed in hypersaline environments, where even slight variations in salinity and pH can dictate osmotic stress and ion toxicity levels, thereby selecting for distinct microbial taxa.

Using metagenomics, our results offer a fresh perspective on the relative abundance of the microbiome of the Khewra Salt Mine. This viewpoint clarifies the microbiome’s functioning, variety, and distribution and how it relates to physicochemical characteristics. Therefore, this study is the first to use metagenomics to investigate the halophiles’ earlier unreported metagenomic community profiles and correlate them with their physicochemical parameters and the native microbiota of the Khewra Salt Mine [69]. The fundamental physicochemical characteristics of the brine and soil under study were most likely only marginally correlated with the observed variations in the makeup of the microbial communities. Soil samples exhibit higher levels of potassium (K) and sulfate (SO_4_), which are essential for the osmotic regulation of Firmicutes and Bacteroidetes, the dominant phyla in soil. Meanwhile, brine contains higher calcium (Ca) concentrations, which may influence the growth of Proteobacteria that often exhibit calcium-binding capabilities [70,71]. The near-neutral pH in brine creates favorable conditions for Proteobacteria and Euryarchaeota, organisms known for their ability to thrive in environments where ionic exchange mechanisms are crucial [72,73]. These specific ion differences support niche specialization, where the ionic composition synergistically interacts with salinity and pH to shape microbial communities. Another study titled “Microbial Assemblages in Pressurized Antarctic Brine Pockets” (2019) investigates the microbial communities within Antarctic brine pockets. The research found that Proteobacteria dominated in both samples, with higher abundance in one of the brine pockets (65.9%) compared to the other (41.1%) due to the pH being almost neutral. This dominance underscores the significant role of Proteobacteria in these environments [73].

This is because there is very little difference in the samples between the two groups, and the soil and brine in the Khewra Salt Mine have nearly identical salinity, pH, and primary ion levels. All of the brines had almost neutral pH values and were almost saturated with NaCl, as reported by Ref. [74] from the Polish Bochnia Salt Mine in Poland, while the pH of soil samples was slightly above. It follows that any observed variations in the microbial composition were most likely caused by the mine’s specific location and other parameters like surface water availability or variations in the trace element content of the soil and brine.

In hypersaline environments such as the Dead Sea, higher salinity and alkaline conditions similarly favor Firmicutes and Bacteroidetes, while near-neutral pH regions support Proteobacteria-rich communities. This pattern underscores the predictive role of salinity and pH in microbial structuring [75,76]. Likewise, the findings from our study of the Khewra Salt Mine mirror well-documented trends in other saline ecosystems, substantiating the claim that pH and salinity gradients are fundamental in shaping microbial diversity.

The 16S rRNA study for both groups found significant variations in the microbiome makeup at several mine locations. Based on beta diversity analysis, which measures the distance between the six samples from six distinct locations in the mine, a statistically significant difference in biodiversity was observed. Similarly, the alpha diversity analysis also observed a difference in the diversity of both groups, with the soil samples showing high microbial relative abundance compared to brine. Because organisms need particular adaptations to survive under different degrees of ionic stress and hydrogen ion concentration, the clustering patterns in beta diversity directly reflect how microbial compositions are structured along the salinity and pH gradients. The mineralization of brine and soil may be connected to these variations [77]. The Ca^2+^ and Mg^2+^ ion concentrations were higher in brine than in soil, while the concentrations of K^+^ ions were higher in soil than in brines, which might impact two distinct segments of the microbial population. These specific ion differences support niche specialization, where the ionic composition synergistically interacts with salinity and pH to shape microbial communities [78,79]. The PCA analysis, which revealed the soil samples to be widely dispersed, supported this observation. Samples of brine were found to be more grouped.

Taxonomic analysis showed that phyla Firmicutes are more frequent in soil samples 1 and A1 from spot 1 of the mine than in those of other spots. The Firmicutes sequence content was 59.39 in sample A1, only 6.72 in brine sample SBK6 from spot 6 (pond 3 draining inside the mine), and the other samples had slight differences in the groups accordingly. The highest occurrence of phyla Bacteroidota was observed in soil samples, with the highest value among three soil samples in 3A from the third spot, 43.63. Meanwhile, the phyla Proteobacteria was dominant in brine samples. This is an intriguing finding because the microorganisms that were found do not belong in a hypersaline environment [33]. This could indicate that several halotolerant organisms frequently found in different environments have contaminated the Khewra Salt Mine. This is conceivable given the volume of visitors and surface water that enters the mine; however, given the brine’s extremely high salinity of more than 30% NaCl, the halotolerant organisms would need to evolve defense mechanisms comparable to those of strict halophiles to survive in such circumstances for an extended amount of time [77].

Our study’s taxonomic findings imply that the high Proteobacteria abundance in brine shown by the 16S rRNA results may represent the actual distribution of these bacteria in the environment. Other researchers have also found significant concentrations of Proteobacteria in brine that they have examined, including brine from the Ciechocinek city’s graduation towers and the Karak Salt Mine [25,80]. Numerous species that sequencing 16S rRNA proved to be extremophiles were found in a large number of samples, such as Proteobacteria, Campilobacterota, Desulfobacterota, or Patescibacteria. This evidence demonstrates that salinity and pH in soil and brine samples of the Khewra Salt Mine correlate directly with the observed microbial compositions. Slight differences in these parameters result in ecological filtering, promoting the dominance of specific taxa. These correlations are consistent with global trends observed in other hypersaline ecosystems, strengthening the argument for the role of salinity and pH in structuring microbial diversity.

The human gut microbiome contains some halophilic and halotolerant microorganisms, and the gut halophilic microbiota is thought to be associated with many chronic illnesses [81]. Likewise, certain studies confirmed an increase in some Firmicutes families (Veillonella and Streptococci) and Proteobacteria and a decrease in some Actinobacteria populations (Bifidobacteria) and some Firmicutes families (Lactobacilli) (*Enterobacteriaceae* spp.) [82]. These results show that the microbial richness that may be involved in the synthesis of amino acids has decreased, which weakens the function of the epithelial barrier and causes intestinal problems (i.e., IBS symptoms) [83]. Our 16S metagenomics findings revealed the maximum richness of Lactobacilli and Bifidobacteria in contrast to the least number of Streptococci and the absence of Veillonella (Firmicutes) followed by *Enterobacteriaceae* spp. (Proteobacteria). Therefore, it can be assumed that considering the microbial composition of the Himalayan Pink Salt, it may not contribute to causing any pathogenic disease while using it domestically.

Samples from the colon, intestine, and faeces of people with obesity, type 2 diabetes, celiac disease, or inflammatory bowel disease have shown higher frequencies of Ruminococcaceae, Rikenellaceae, Desulfovibrionaceae, *Akkermansia muciniphila*, and potentially pathogenic Bacteroides and *E. coli* than those from healthy persons [84]. Many other diseases, including obesity, have a pathogenesis that the gut microbiota may influence. Our study has observed no causative abundance of these species as susceptible. The microorganisms involved in infections caused by food are mainly *E. coli*, *Salmonella*, and *Staphylococcus* [85] phylum Pseudomonadota and Bacillota; accordingly, in our analysis, we could not found such find any pathogenic species or relatively high abundance of any species which may contribute to causing the aforementioned dietary disorders.

## 5. Conclusions

This investigation marks a groundbreaking step in comprehending the halophilic microbial communities thriving in the unexplored terrain of Pakistan’s Khewra Salt Mine. Our research reveals a remarkably diverse microbiome within the mine, teeming with numerous potentially unidentified bacterial species. The prevalence of halophiles bolsters the resilience of these bacteria in such a challenging environment. Our analysis identified archaeal and bacterial communities such as Firmicutes, Halobacterota, Desulfobacterota, Campilobacterota, Patescibacteria, and Proteobacteria residing in the Khewra Salt Mine. While our exploration unveiled no abundant pathogenic species that could pose health risks, the uncharacterized OTUs represent a substantial portion of the overall population, hinting at an intriguing diversity not yet fully understood. The discovery of novel genera underscores the potential for valuable bioproducts, urging a more comprehensive characterization effort. Future investigations, including diverse sampling points such as halite crystals from distinct sediment layers and periodic assessments across the mine, promise more profound insights into the ecology of extreme halophiles. Our forthcoming plans involve a thorough diversity analysis to paint a more comprehensive portrait of microbial variety. Given the limitations of metagenome analysis in deciphering gene expression and protein synthesis, a meta-transcriptomic approach becomes imperative to unravel the functional intricacies of these microbial ecosystems. Additionally, employing meta-transcriptomics offers a promising approach to unravel functional complexities of microbial ecosystems. This method could address specific confusions such as gene expression profile differences between soil and brine microbial communities in response to salinity and pH variations, the metabolic pathways that enable microbial survival in high-salinity and variable pH conditions, the identification of specific gene products or pathways linked to osmotic stress, ion transport, and enzymatic activity for biotechnological applications, and the presence of novel biosynthetic gene clusters that could lead to the discovery of new antimicrobials or other bioactive compounds.

Moreover, considering the resemblances between halites on Mars and terrestrial environments, exploring comparable environments on the Red Planet could unveil preserved organic matter and potential biomarkers. Research on microbial resilience in high-salinity environments, such as the Atacama Desert and Antarctic brine pockets, supports the idea that life could persist in subsurface briny reservoirs on Mars or icy moons like Europa and Enceladus [73]. The ability of halophiles to withstand extreme osmotic pressure, radiation, and desiccation suggests that such microorganisms, or their biosignatures, may be detectable in future astrobiological explorations [86]. Studies have further demonstrated that microbes, such as *Planococcus halocryophilus*, can thrive in chloride and perchlorate brines, which are commonly found on Mars, offering a promising model for understanding microbial life in briny environments outside Earth [71]. Moreover, microbial diversity in hypersaline environments, such as the Ntwetwe Pan of Botswana, provides terrestrial analogs for understanding microbial life in Martian playa deposits [10]. These studies suggest that similar microbial communities may exist in Martian salt deposits, where they could provide insights into ancient life forms or even potential extant life. Given these connections, further investigation into the extremophiles of the Khewra Salt Mine could yield crucial data for planetary science, particularly regarding the potential for microbial life to endure in extraterrestrial hypersaline environments. Future studies integrating metagenomic and meta-transcriptomic approaches may provide deeper insights into the metabolic pathways that enable microbial survival in high-salinity conditions, refining models for life detection beyond Earth [74]. Such findings, potentially concentrated by evaporative processes, might signify the preservation of life over extensive geological timescales. The uncharted microbial diversity of the Khewra Salt Mine presents a fertile ground for future studies. Leveraging advanced omics approaches and integrating them with geochemical analyses will provide deeper insights into microbial functionality and ecological significance. Such efforts promise to uncover novel resources with implications for biotechnology, astrobiology, and environmental science.

## Figures and Tables

**Table 1:** Physicochemical characteristics of soil samples, including sample pH, salinity, moisture content (%), conductivity (mS/mL), organic matter, and concentration of selected ions and metals (in wt%, Fe in mg L^−1^).

Soil Samples	Loc.	pH	Salinity	Moisture Content	Conductivity	Na	K	Cl	Ca	Mg	Fe (mg/L)	SO_4_	Nitrogen Content	Total Organic Carbon	Organic Matter
1A	SWDKM	7.9	30%	26.59%	95	27.2	0.34	42	0.018	0.053	9.51	3.2	0.11%	0.29%	1.01%
2A	SSHWKM	8.2	32%	10%	95.5	28.6	0.36	44.5	0.04	0.059	15.5	0.35	0.38%	0.35%	0.95%
3A	SCFMKM	8.65	36%	8.27%	99	33.5	0.42	49.4	0.041	0.065	16.8	0.32	0.97%	0.39%	1.05%
MEAN		8.25	0.327	0.149533	96.5	29.8	0.39	45.3	0.033	0.059	13.9	1.29	0.0049	0.00343	0.010033
SD		0.38	0.031	0.101147	2.1794	3.31	0.04	3.764	0.013	0.006	3.88	1.654	0.0044	0.0005	0.000503

SWDKM = Soil near water drips coming from the roof of the Khewra Salt Mine spot 1. SSHWKM = Soil attached with salt halite from walls of the Khewra Salt Mine spot 2. SCFMKM = Soil from cutting face of mining area inside the Khewra Salt Mine spot 3.

**Table 2:** Physicochemical characteristics of brine samples, including sample pH, salinity, moisture content (%), conductivity (mS/mL), organic matter, and concentration of selected ions and metals (in wt%, Fe in mg L^−1^).

Brine Samples	Loc.	pH	Salinity	Conductivity	Na	K	Cl	Ca	Mg	Fe (mg/L)	SO_4_	Nitrogen Content	Total Organic Carbon	Organic Matter
SBK1	P1KSM	7.25	32.75%	108	22.3	0.126	44.85	0.321	0.097	3.28	0.11	0.10%	0.10%	0.67%
SBK2	P2KSM	6.8	29.90%	100	19.1	0.132	40.33	0.309	0.085	4.59	0.8	0.07%	0.27%	0.49%
SBK3	P3KSM	7.5	33.30%	105	31.31	0.138	50.24	0.342	0.112	6.24	0.2	0.08%	0.15%	0.37%
Mean		7.183	0.319833	104.3333	24.237	0.132	45.14	0.324	0.098	4.7033	0.37	0.000823	0.001733	0.0051
SD		0.355	0.018251	4.041452	6.3312	0.006	4.9614	0.0167	0.014	1.4833	0.3751	0.000172	0.000874	0.00151

P1KSM = Pond 1 in the Khewra Salt Mine. P2KSM = Pond 2 in the Khewra Salt Mine. P3KSM = Pond 3 in the Khewra Salt Mine.

## Data Availability

Additional data for 16S rRNA sequences of isolates in this study can be found at https://www.ncbi.nlm.nih.gov (accessed on 24 November 2024) with accession no. PRJNA1058869.
